# “The targets…are driving the agenda and that probably needs to change”: stakeholder perspectives on HIV partner notification in sub-Saharan Africa

**DOI:** 10.1186/s12889-023-17422-9

**Published:** 2024-02-19

**Authors:** Maureen McGowan, Kate Bärnighausen, Astrid Berner-Rodoreda, Shannon A. McMahon, Caroline Mtaita, Joy Mauti, Florian Neuhann

**Affiliations:** 1 Heidelberg Institute of Global Health, Heidelberg University, Im Neuenheimer Feld 130.3, 69120 Heidelberg, Germany; 2 School of Public Health, University of the Witwatersrand, Johannesburg, South Africa; 3 Department of International Health, Social and Behavioral Interventions Program, Johns Hopkins Bloomberg School of Public Health, Baltimore, USA; 4 School of Medicine and Clinical Sciences, Lewy Mwanawasa Medical University, Lusaka, Zambia

**Keywords:** HIV, Contact tracing, Partner notification, Sub-Saharan Africa, Multi-level analysis

## Abstract

**Background:**

Voluntary assisted partner notification (VAPN) in HIV contact tracing is a globally recommended strategy to identify persons who have been exposed to HIV and link them to HIV testing and follow-up. However, there is little understanding about how VAPN is experienced by stakeholders in sub-Saharan African (SSA) contexts. We conducted a multi-level and multi-national qualitative analysis evaluating stakeholder perspectives surrounding VAPN implementation to inform the development of future VAPN policies.

**Method:**

We conducted in-depth interviews (IDIs) with VAPN stakeholders at global (*n* = 5), national (*n* = 6), and community level (*n* = 4) across a total of seven SSA countries. Eligible participants were ≥ 18 years old and had experience developing, implementing, or overseeing VAPN policies in SSA. We sought to understand stakeholder’s perspectives on policy development, implementation, and perceived outcomes (barriers and facilitators). Interviews were audio recorded, transcribed, and analyzed thematically using a combination of inductive and deductive approaches.

**Results:**

Between December 2019 and October 2020 we conducted 15 IDIs. While participants agreed that VAPN resulted in a high yield of people newly diagnosed with HIV; they noted numerous barriers surrounding VAPN implementation across global, national, and community levels, the majority of which were identified at community level. Barriers at global and national level included high target setting, contradictory laws, and limited independent research disenfranchising the experiences of implementing partners. The barriers identified at community level included client-level challenges (e.g., access to healthcare facilities and fear of adverse events); healthcare worker challenges (e.g., high workloads); limited data infrastructure; and cultural/gender norms that hindered women from engaging in HIV testing and VAPN services. In response to these barriers, participants shared implementation facilitators to sustain ethical implementation of VAPN services (e.g., contact tracing methods) and increase its yield (e.g., HIV self-testing integrated with VAPN services).

**Conclusion:**

Overall, stakeholders perceived VAPN implementation to encounter barriers across all implementation levels (global to community). Future VAPN policies should be designed around the barriers and facilitators identified by SSA stakeholders to maximize the implementation of (ethical) HIV VAPN services and increase its impact in sub-Saharan African settings.

**Supplementary Information:**

The online version contains supplementary material available at 10.1186/s12889-023-17422-9.

## Background

While voluntary assisted partner notification (VAPN) is globally recommended as a feasible and effective strategy for HIV contact tracing [1–3]; little is known about how the implementation of VAPN is experienced by policymakers and implementers in sub-Saharan Africa (SSA). VAPN is a long-established practice in the United States and Europe [3–5] and has more recently been adopted into national guidelines in the SSA region following World Health Organization (WHO) recommendation in 2016 [1, 6, 7]. The aim of VAPN is to reach sexual and/or drug injecting partners as well as biological children of persons recently diagnosed with HIV (the index client), and to provide opportunities for HIV testing and early linkage to prevention (e.g., pre-exposure prophylaxis [PrEP]) or treatment (e.g., antiretroviral therapy [ART]) services [1, 7–10]. Although sub-Saharan Africa has made notable strides in addressing HIV [11, 12]; VAPN has been suggested as an additional cost-effective strategy [13, 14] to reach the estimated ~ 2.4 million people [15–17] living with undiagnosed HIV infections in the region and to achieve the UNAIDS 95–95-95 targets by 2030^1^ [18, 19]. Recent studies in sub-Saharan Africa often focus on client-level experiences and highlight that VAPN is largely acceptable, feasible, and effective at tracing partners exposed to HIV [6, 9, 10, 20–24]. VAPN has also been found to be appropriate for targeting hard-to-reach populations (e.g., men) for testing [10, 25–27] and is often reported to have insignificant adverse events (e.g., intimate partner violence [IPV]) [1, 6, 10, 22, 28]. Still, numerous logistic and ethical concerns about its implementation have been raised including patient confidentiality; risk of involuntary HIV status disclosure; appropriateness of VAPN for key and vulnerable populations (e.g., men who have sex with men [MSM], female sex workers [FSWs] [29–32]; youth [31, 33]; refugees [26, 34], and incarcerated populations[35]); client concerns about adverse events; and fear of community stigma and discrimination [25, 26, 28, 34, 36, 37]. Studies that included female index clients specifically highlighted fear of gender-based violence following HIV status disclosure as a primary barrier to engaging with VAPN services [26, 30, 37, 38].

Additionally, some studies have investigated the perspectives of healthcare workers (HCWs) delivering VAPN services at community level [25, 26, 32, 36, 37]. While HCWs in these studies often perceived VAPN to be an appropriate HIV testing strategy for their clients [25, 26, 28, 32], they raised concerns about a perceived need for specialized VAPN training [39]; high workloads and resources necessary to conduct partner tracing [25, 26]; a perceived lack of self-efficacy delivering VAPN services [36]; and expressed fear of violence providing services at community level [25, 26, 36]. A major barrier also raised by healthcare providers was limited awareness of VAPN services at community level—and among some healthcare workers—thereby leading to missed opportunities for HIV testing [25, 28]. However, only few studies have aimed to understand perspectives surrounding VAPN implementation beyond community level (i.e., client and healthcare level) by including views from national stakeholders [40, 41]. To date, little is understood about how VAPN is perceived across global, national, and community levels.

Our aim was to understand perspectives from global, national, and community stakeholders and to capture variability in VAPN implementation experiences across countries by including participants from Malawi, Sierra Leone, South Africa, Kenya, Zimbabwe, Tanzania, and Eswatini. We specifically aimed to identify barriers to HIV VAPN implementation and to document both practiced and proposed facilitators to support its ethical implementation and yield. Findings from this study may inform the development of future HIV VAPN guidelines to reflect context-specific challenges in sub-Saharan Africa, to increase VAPN yield (i.e., number of people identified as HIV-positive through VAPN services), and to maximize the ethical implementation of VAPN.

## Methods

### Study setting

Qualitative data were collected online with global, national, and community stakeholders. The sub-Saharan African region was selected for this study because 1) it is the most heavily affected region by HIV/AIDS globally [42] and 2) several countries in the region adopted VAPN strategies following global recommendation. To the best of our knowledge, this is the first study to investigate VAPN across all three levels of implementation as well as across implementing countries.

### Study design & sampling

We conducted semi-structured in-depth interviews (IDIs) among male and female stakeholders who were $\ge$ 18 years old and had previous experience developing, implementing, or overseeing HIV VAPN guidelines in sub-Saharan Africa either at global, national (e.g., governing bodies, non-governmental organizations [NGOs], implementing organizations), or community (e.g., healthcare worker, service provider) levels. National and community stakeholders were found eligible if they could speak to VAPN in the SSA region beyond their individual communities. Authors KB, ABR, and FN recruited study participants through the purposive sampling of professional contacts who were engaged in HIV work. Of the potential study participants who were contacted (*n* = 22), 68% (*n* = 15) were deemed eligible by their function, role in HIV programmes, and/or implementation of VAPN, and agreed to study participation. Thus, the countries represented in this study- at national and community level- were a convenience sample. We considered the sample size (*n* = 15) sufficient [43] for this study based on the depth and breadth of the themes discussed in the interviews. The richness of the data gave us confidence that significant themes and perspectives have been adequately analysed. Prior to the interview, ABR and KB provided an overview of the study (i.e., study cover letter, study information sheet) to potential interview participants. After an initial positive response, we re-contacted potential participants and provided them a consent form to be voluntarily signed for study participation. Ethical approval for this study was granted by the ethics committee of the medical faculty (*“Ethikkommison der medizinische Fakultät”*) at Heidelberg University (S581/2019).

### VAPN strategies

In this study, we defined VAPN in accordance with the 2016 World Health Organization (WHO) guideline on HIV partner notification [1]. Index clients (i.e., those recently diagnosed with HIV) may voluntarily select one of the following services: *provider referral* (i.e., healthcare providers directly and anonymously contact partners), *contract referral* (i.e., a time-bound contract within which the index client has the opportunity to independently notify partners of HIV exposure and refer them to testing, followed by provider referral if not achieved in the specified time frame), or *dual referral* (i.e., index clients disclose their HIV status alongside the support of providers). The index client may also opt to disclose their HIV status and encourage partners to test independently (without the provision of provider support), referred to as *passive notification.* In this study, we included perspectives on VAPN services as well as passive notification. All forms of VAPN service delivery and passive notification are intended to be implemented voluntarily, in cases without evident risk of IPV, and in alignment with the WHO’s 5 C’s (consent, confidentiality, counselling, correct results, and connection) [44, 45].

### Data collection

Authors MM and ABR, researchers with graduate-level qualitative training, developed three versions of the interview guides in correspondence to stakeholder level- global, national, and community level, respectively (Additional file 1). Interview guides were pilot tested with and approved of by the study team. IDIs collected participant demographics (e.g., gender, age, work experience) and explored themes of VAPN policy development, implementation experiences, perceived outcomes (i.e., benefits, adverse outcomes, yield), and ethical implications. At the time of this study, we did not ask participants about their HIV status nor whether they received VAPN services themselves, as this was beyond the scope of this study. All interviews were conducted by authors ABR (*n* = 10) and KB (*n* = 5), both of whom are experienced qualitative researchers and have spent more than a decade working in sub-Saharan African contexts. Interviews were conducted in English and in a private setting, via an online platform of the participant’s choosing (e.g., Skype), audio recorded with participant consent, transcribed verbatim, and analysed via Nvivo version 12 (QSR International, Melbourne, Australia).

### Data preparation & analysis

We analysed demographic data using descriptive statistics and analysed qualitative data using the principles of Thematic Analysis (TA) [46] which included a combination of inductive and deductive approaches; the latter of which was informed by a theoretical framework by Turcotte-Tremblay et al. (2017) used to measure innovation outcome anticipation and desirability [47]. Author MM (with consensus of the study team) classified VAPN implementation practices which were unanticipated and undesirable (in contrast to practices anticipated by global VAPN guidelines [1, 44, 48, 49]) as “barriers”. VAPN practices which were unanticipated (in contrast to global guidelines) but desirable, were referred to as “facilitators”. This framework was deemed appropriate to frame our analysis through its recent adaptation to measure unintended uses and consequences of a peer-delivered HIV prevention intervention among female sex workers in East Africa [50].

TA was conducted in accordance to a six-stage process defined by Braun and Clarke (2006) [46]. Authors MM, KB, and ABR familiarized themselves with the interview transcripts by reading and re-reading the data. MM and KB then inductively coded transcripts in sets of five to discuss converging or diverging codes. Codes were presented to the larger study team, discussed, and agreed upon via group consensus. Themes identified as “barriers” were then mapped onto a multi-level cascade [51] to indicate the implementation levels at which these barriers occurred. Thereafter, themes identified as “facilitators” were separated into two overarching domains, namely, aims to 1) sustain ethical VAPN implementation and 2) increase VAPN yield.

## Results

We conducted 15 in-depth interviews with stakeholders between December 2019 and October 2020, Table 1. Interviews were conducted among global (*n* = 5), national (*n* = 6), and community stakeholders (*n* = 4) and lasted, on average, 53 min. Two-thirds of participants were male (*n* = 10) and one-third was female (*n* = 5). Participants had a median age of 43 years (interquartile range [IQR] 37–46.5 years). National stakeholders represented perspectives from Malawi, Sierra Leone, South Africa, Kenya, and Zimbabwe. Community stakeholders represented perspectives from Malawi, Tanzania, and Eswatini. Most participants had extensive work experience in the field of HIV with 64% (9/14) reporting a decade or more of experience (range 2 to > 30 years).

**Table 1:** Demographic characteristics of participants

Demographics	Participants (*n* = 15)
Gender (n, %)	
*Female*	5 (33%)
*Male*	10 (67%)
Age^a^ (median, IQR)	43 (37–46.5)
Years worked in the field of HIV^b^ (median, IQR)	
*0–5 years*	3 (21%)
*6–10 years*	2 (14%)
*11–15 years*	5 (36%)
> *15 years*	4 (29%)
VAPN implementation level (n, %)	
*Global level*	5 (33%)
*National level*	6 (40%)
*Community level*	4 (27%)
Country of VAPN implementation at national and community level^c^ (n, %)	
*Malawi*	3 (30%)
*Tanzania*	2 (20%)
*Sierra Leone*	1 (10%)
*South Africa*	1 (10%)
*Kenya*	1 (10%)
*Zimbabwe*	1 (10%)
*Eswatini*	1 (10%)

*Abbreviations*: *IQR* interquartile range, *VAPN* voluntary assisted partner notification

^a^As reported by 73% of participants (*n* = 11)

^b^As reported by 93% of participants (*n* = 14)

^c^As reported by 67% of participants (*n* = 10)

**Fig. 1:**
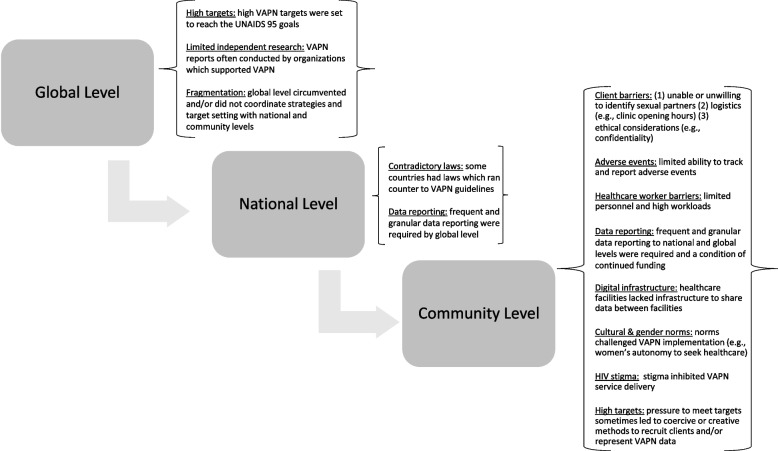
Barriers to VAPN implementation across implementation levels, as identified by VAPN stakeholders (*n*=15)

Below we summarize the identified barriers to VAPN implementation, as reported by participants. These barriers followed a top-down cascade mirroring the implementation of VAPN policy from global to community levels, Fig. 1. Thereafter we present both practiced and proposed facilitators to support VAPN implementation in terms of ethical implementation and yield.

### Global level: setting high global targets challenges ethical VAPN implementation

Although most global stakeholders expressed support towards VAPN implementation as a means to identify those exposed to HIV and link them to care; stakeholders across all implementation levels identified *“pressure” (community-Tanzania, male, participant 14)* to reach targets set at global level as a major barrier. Participants across national and community implementation levels (and one global-level participant) identified some of these targets as 1) number and proportion of elicited partners’ contact information; 2) partners initiated HIV testing; 3) partners tested HIV-positive; and 4) partners linked to care. These participants described that target setting had at times *“overstepped” (national- South Africa, male, participant 10)* ethical boundaries and compelled coercive practices:

*…they [PEPFAR] have set these really high targets…. [target setting] that’s really pushing people to more coercive methods, kind of pressurizing people to share information about their partners and that’s really damaging to the future of the program… I think we are really at an important crossroads right now to ensure that it’s a service to a person with HIV and their partners. It’s not a target setting activity (global, female, participant 4)*

Ethical implementation was particularly challenging in contexts where subsequent funding and grants were dependent on VAPN target performance:

*…I mean, the grant is based on delivery of and their performance against these targets. So, it's performance against the [UNAIDS] 90s targets and then there's sub-indicators there... one of them is proportion of index testing. So, there's a financial incentive responsibility linked to it. (national- South Africa, male, participant 10)*

*…all community-based [services] and NGOs [non-governmental organizations] as long as we are funded by the government…If you are funded by PEPFAR, our PEPFAR funding comes in two major settings like the USAID and CDC. So, anybody who is funded by those two major organizations, they have to do PNS [partner notification services]. And we have targets for PNS. (national-Kenya, female, participant 11)*

An additional challenge raised by some participants was inconsistency between global recommendation, national policy translation, and implementation on the ground. A participant from Tanzania described this challenge as follows:

*…there was a lag period [when VAPN was introduced] between the government green light and the introduction of index testing [VAPN]. So, at that time, like, every [implementation] partner who was getting funding from PEPFAR was improvising like how to capture this information. So, it differed actually from one place to another place. (community- Tanzania, male, participant 1)*

Further challenges at global level surrounded the dissemination of VAPN outcomes. Some participants expressed that the limited publication of VAPN studies conducted by independent researchers (not affiliated to major VAPN supporting organizations) was biased to reflect positive outcomes of VAPN services and thereby disenfranchised the experiences of implementing partners:

*…our data is no longer trustworthy. And so, we have this echo-chamber effect that whatever gets dictated for the implementing partners, gets turned into success, so it’s quite difficult to make any kind of decision on the base of routine program data anymore, and you see, even in the published peer reviewed literature it’s very biased evidently. If you look at the funding source for almost any of those studies, it’s related to PEPFAR. (national- Malawi, male, participant 9)*

### National level: contexts with contradictory laws fragment VAPN implementation

A primary barrier identified at national level by participants, was when VAPN implementation guidelines contravened national laws. Laws criminalizing the transmission of HIV^2^; laws withholding the provision of HIV services to persons unwilling to disclose partners’ contacts; laws criminalizing key populations (e.g., MSM,^3^  FSWs^4^),  and (some) data privacy laws ran counter to the tenets of VAPN. For example, national data privacy laws at times prohibited the sharing of client data between healthcare workers, which hindered the delivery of contact tracing services. A participant highlighted this unresolved paradox between VAPN guidelines and the National Communications Act in Tanzania:

*… if I am working at the facility, I am a doctor, I saw you today. We discussed [VAPN] and you offered the [phone] number because you started [HIV] treatment and because I needed your contacts…And then I take that number and give it to a nurse. So basically, I did not ask your consent to give your number to somebody else. So, it somehow led to some issues in that context. (community - Tanzania, male, participant 14)*

A global stakeholder similarly described challenges implementing VAPN in contexts with laws criminalizing the transmission of HIV. This participant expressed the challenges this poses to healthcare providers delivering VAPN services as well as clients receiving the service:

*…you are going to have countries where HIV transmission is criminalized and that sort of does put you in a difficult situation and puts the [healthcare] provider in a difficult situation. So, you need to be really clear about what the law of the land is, like what the patient’s rights are, what their partner’s rights are, so I think that needs to be really clear to people. And then I think you have got things like medical secrecy laws and confidentiality, which are explicit, which then depending on what those criminalization laws are and other policies are, can kind of supersede confidentiality and patients may not know that. (global, female, participant 4)*

Challenges implementing VAPN in countries with laws criminalizing or prosecuting HIV transmission is also mirrored by a national stakeholder in Sierra Leone who described that perceived fears of prosecution dissuaded clients from disclosing their partners’ contacts—particularly among key populations:

*There’s a law that talks about “willful infection”, that means if you willfully, if you know that you are HIV-positive and then you sleep with another person that is HIV-negative and that person ends up getting HIV, you are supposed to be prosecuted and you are supposed to be punished. But of course, most of the time, people don’t come out [disclose contacts]” (national-Sierra Leone, male, participant 8)*

### Community level: ethical concerns, cultural norms, and limited resources challenge VAPN implementation on the ground

Participants reported that most barriers to VAPN implementation occurred at community level. Participants often reported these challenges at client-level in which clients and/or their partners were at times unwilling or unable to engage in HIV testing or notification services. For example, participants reported logistical challenges engaging with HIV testing services: “*working hours [of the HIV clinic] are when men are at work” (community-Tanzania, male, participant 14)* or reflected clients’ ethical concerns surrounding confidentiality or fear of involuntary HIV status disclosure- as evidenced by clients providing inaccurate partner contact information. Some study participants confirmed that clients’ ethical concerns were valid in which they observed (albeit rarely) cases in which client confidentiality had been broken and VAPN underpinned partnership dissolution, intimate partner violence, and even suicide. Some participants highlighted that these adverse events were rarely reported because 1) follow-up reporting tools to measure adverse events among index clients and their partners were often not required nor standardized and 2) personnel shortages inhibited follow-up services:

*…. it was a reminder that the PEPFAR policy for VAPN implementation [which] included a detailed interview for intimate partner violence screening, as well as a follow-up after contacts were tested with the index case. And of course, that makes the implementation even more unrealistic because it means that you have to make another follow-up, an active follow-up with the index after sometime once the contacts have been tracked down. So, it seems utterly unrealistic to try and do that (national-Malawi, male, participant 9)*

Similarly, participants described carrying out VAPN services as generally challenging for healthcare providers due to “*staff shortages” (community-Tanzania, male, participant 14)*, perceptions that VAPN was *“very labor-intensive” (national-South Africa, male, participant 10)* and that it conflicted with other work responsibilities. Similarly, few healthcare workers were available to conduct *“granular and elaborate” (national- Malawi, male, participant 9)* VAPN data reporting: *“I think they are over eight hundred, close to nine hundred data elements [are] to be reported [to PEPFAR] from every facility for every quarter from the testing program alone” (national- Malawi, male, participant 9)*. Challenges recording data was then exacerbated due to fragmentation within and across data systems (i.e., digital versus analog) as reported by a global stakeholder, who accounted cases in which VAPN reporting was made on patient notes which then need to be *“extracted and de-identified”* leading to lack of *“synchronicity between reporting systems” (global, male, participant 7)*. Limited available human resources and inefficient digital infrastructure were also highlighted by participants as barriers to tracing index clients and their partners across healthcare facilities to follow-up whether they had initiated HIV testing and/or care:

*“… [the contact is] identified at Facility A but maybe they could go to Facility B for HIV testing. So, in this case, if you don’t have any documentation which could support this, then you would just get it from the [word of] mouth to say I got tested here and there” (community-Malawi, male, participant 2)*.

In addition to client and provider level barriers, community-level cultural understandings and gender norms at times further hindered the implementation of VAPN. This challenge was raised by stakeholders in contexts with rigid gender norms where it may be viewed as inappropriate for women to seek healthcare services (e.g., HIV testing) without male accompaniment: *“…in Eswatini, a man should lead in anything. So, if she [female partner] wants her[self] to go to [HIV] test, she should have consulted first with him [male partner] before going to test or even possibly…they can go hand-in-hand which is also difficult at times” (community-Eswatini, female, participant 15).* Similarly, contexts where promiscuity or engagement in multiple partnerships is a norm- yet found unacceptable- clients may refuse HIV testing and contact tracing services due to a fear of being blamed for HIV transmission and perceived subsequent adverse effects. Many participants described that community-level stigma of HIV was a continuous challenge for implementing HIV testing and VAPN. The fallout from stigma was evidenced in cases wherein clients were *“afraid to be scrutinized” (national-Tanzania, male, participant 8*) by medical personnel for being non-adherent to ART and therefore re-tested and re-registered as a new index client in a different healthcare facility. Further, participants across several countries described how HIV stigma and challenges providing VAPN services proved particularly challenging in small rural communities- where the healthcare workers know everyone in the community and may try *“to warn other people. Like stay away from that person [index client]” (community-Tanzania, male, participant 1)* or when HCWs themselves may become part of the contact tracing chain:

*Like a health worker’s husband is going to be listed as a contact eventually. Because there is only so many men in [the] community, and HIV is being transmitted somehow. (global, female, participant 4)*

Finally, a major barrier identified by participants at community level mirrored the primary barrier at global level, namely, reaching VAPN targets. Participants described that implementing partners felt pressured to maintain an unyielding focus on meeting targets within pre-determined time-frames: *“…make sure that within that month that person [index client] mentions his or her sexual partner” (community-Tanzania, male, participant 1)*. This, participants said, could lead to a coercion of clients to accept VAPN services. In extreme cases this scenario entailed calling partners for whom index clients had not given consent or reporting clients who had been *“poached” (national-Malawi, male, participant 9)* from ART clinics and listing them as new patients at a different health facility:

*…you have pressure for targets, and you already know that this client is [HIV] positive and most likely if they give me their sexual partners most likely I'll be able to get HIV-positive clients and add up to [reach] my target, then there are people [healthcare workers] who will call people [partners of clients] who do not give the consent of being called which is very annoying. (national-Kenya, female, participant 11)*

In addition to coercion, a few participants described creative data reporting mechanisms to increase the reported yield of VAPN (i.e., number of persons tested HIV-positive). A national stakeholder from Malawi described how the denominator of the data was adjusted in which known HIV-positive partners of index clients were recorded as newly-elicited contacts:

*…whenever there was a person recorded who was HIV-positive, who reported a [HIV] positive partner, that record was abstracted and interpreted as a successful VAPN client… So, it was a complete reversal of the [VAPN] policy but of course that yielded very high positivity rates because, of course, you can imagine that if you are turning the entire strategy on its head, and you are starting with a [HIV-] positive [client] and you are calling that a contact, and of course this person came completely on their own steam [accord] but they happen to report a [HIV] positive partner. Then you can concentrate the [HIV] positives [denominator] amongst the that small sub-group you are eliciting (national-Malawi, male, participant 9)*

### Stakeholder strategies facilitate ethical and impactful VAPN implementation

#### Sustain ethical implementation

In response to the identified barriers surrounding VAPN implementation, participants described enacted solutions—predominately at community and national levels-which aimed to sustain ethical implementation of VAPN and increase its yield, as highlighted in Table 2. Regarding strategies aimed at sustaining ethical implementation, some participants described adapting global VAPN policies to local contexts, typically through discussions with relevant stakeholder groups and implementing partners. Tailored VAPN services were also recommended to be carried out by healthcare workers who demonstrated HIV counselling experience and who received specialized VAPN training.

Further, participants across countries described implementing creative methods to ensure the safety (i.e., confidentiality and anonymity) of an index client and/or partner(s) at community-level. One such method was to implement a *“routine health campaign” (community-Tanzania, male, participant 1)* to disguise the targeting of a specific household for HIV testing by providing health screenings (i.e., non-communicable disease screenings) to numerous homes in a community. Another method, described by a participant from Eswatini, was to ask an index client about all persons who may have been exposed to HIV including, *“all sexual partners, biological children, family members and associates and family. And so, in our associates, we have friends, colleagues and all” (community-Eswatini, female, participant 15)*. The participant described that this method upheld the ethical implementation of VAPN services in which index clients could list sexual partners as *“associates”* thereby allowing the client to maintain confidentiality of the nature of their relationship. Additionally, a participant from Kenya described anonymously reaching sexual partners by referring to community sensitization projects:

*“… the good thing is that we have adverts all over, even in Kenya, and even we have a current advert about HIV-testing that is on the TV, so we were able to say, you know, it’s important that you get to know your HIV status. That's why you can see some advertisements about people even getting to do [HIV] self-tests. We are reaching to you to see, if you haven't known your HIV-status, whether you would want us to give you a free service, and we can either come to where you are and give you the service or you can come back to us and seek the service.” (national-Kenya, female, participant 11)*

In addition to creative contact tracing methods to support ethical contact tracing, some participants described methods to prevent involuntary HIV status disclosure of the index client. The first method, described by participants from numerous countries (Malawi, South Africa, Kenya, Tanzania), included pretending not to know an index client when they returned to a health facility with their partner to allow both individuals to be tested for HIV simultaneously. The second method described by a community-level participant from Tanzania was to ensure that an index client and their partner(s) were not scheduled for follow-up ART appointments at the same time. Beyond strategies implemented by healthcare providers at community level, a facilitator reported by a global participant was to promote ethical VAPN services (particularly in rural tight-knit communities) through the rotation of healthcare workers to more distal communities. This rotation system was perceived to mitigate ethical conundrums (i.e., involuntary HIV status disclosure) in areas where HCWs knew most community members. This participant further described the provision of psychological support to HCWs as an aid to promote ethical implementation of VAPN services. Some participants also reported utilizing solutions to maintain data safety and to track adverse events associated with VAPN. For example, participants from Malawi described keeping medical records in *“lockable cabinets” (community-Malawi, male, participant 2)* or recording the contact information from index clients and partners on separate *“ledgers” (national- Malawi, male, participant 9)* to blind anyone (including HCWs) from linking index clients with their partners. An additional facilitator described by a participant working in a community level healthcare facility (in Malawi) described implementing a liability agreement between the client and healthcare facility in the event of a confidentiality breach. Finally, a few participants described that their countries implemented *“hotlines” (national-Zimbabwe, female, participant 13)* or *“surveys and exit interviews” (global, female, participant 4)* for index clients to report adverse events attributed to VAPN services.

#### Increase uptake of VAPN services and VAPN yield

In respect to strategies practiced by participants to increase the yield (or impact) of VAPN services (Table 2); some participants described sensitizing both HCWs and the community to VAPN guidelines and services through manuals, posters, or pamphlets. A participant from South Africa also described the use of *“community champions” (national-South Africa, male, participant 10)* through social media channels to encourage individuals to engage in HIV testing (and if appropriate, VAPN services).

**Table 2:** Facilitators practiced by participants to support the implementation of VAPN services in terms of ethical implementation and yield, respectively (*n* = 15)

Facilitator Domains:	Practiced Solutions:	Quotes:
**Ethical Implementation**	**Developing context-specific and client-centered VAPN policies and guidelines**	*“With the assistance of our funders CDC, PEPFAR…they have global best practices on what has worked in other countries and settings…we take that and also adopt it and adapt it to our national setting. Eswatini, we have our own culture, where they get evidence from, they also have their own culture.”* (community-Eswatini, female, participant 15)
	**Involving relevant stakeholder groups into VAPN policy development discussions**	*“…so, when Kenya started developing [a VAPN policy], we ensured that everyone was put together including the human rights organizations.”* (national-Kenya, female, participant 11)
	**Selecting and training specialized counsellors to conduct VAPN**	*“So, we realize that it's not everyone… PNS [partner notification services] is not for every counsellor. It depends on the age… It depends on the years of experience and the maturity of the counsellor.”* (national-Kenya, female, participant 11)
	**Implementing creative VAPN techniques to ensure confidentiality and anonymity** (e.g., developing routine health campaigns with integrated VAPN services)	*“…we [healthcare workers] are coming door-to-door. We are having like a health campaign, we will test everybody. We will test maybe for blood sugar, we test maybe for non-communicable diseases but also, we test for HIV. So, when they [HCWs] test that person, he will see like okay, it was just a routine campaign. So, when they find him to be [HIV-] positive, they will record him but actually that person wouldn’t know that he was targeted.”* (community-Tanzania, male, participant 1)
	**Implementing methods to prevent involuntary HIV status disclosure**	*“… most of the healthcare providers, they are always very careful that when they schedule appointments to make sure that those [medication] appointments are not scheduled on the same time.”* (community-Tanzania, male, participant 1)
	**Rotating rural HCWs to distal healthcare facilities to prevent involuntary HIV status disclosure**	*“…they [country] were exploring actually moving health workers around. So that the people doing partner services weren’t working in that community, so it would kind of give them one more step of distance which was really potentially helpful for them because they have such small tiny communities.”* (global, female, participant 4)
	**Providing psychological support for HCWs implementing VAPN services**	*“We always used to have like field investigator meetings you know to…go over what happened because you [HCWs] are dealing with a lot of intense psychological stuff. And you need to talk to somebody who can be that external perspective to say, ‘Oh, we understand what you are dealing with, here is what you need to do about it, we can talk about it again tomorrow’.”* (global, female, participant 4)
	**Enhancing data safety (**i.e., using methods to keep medical data safe and confidential)	*“…people had signed a confidentiality agreement form which was like an agreement between the provider and the facility which is providing the [VAPN] services. Just to ensure that once it [confidentiality] is broken, somebody should be liable for that.”* (community-Malawi, male, participant 2)
	**Identifying adverse events of VAPN implementation** (e.g., hotlines and exit surveys)	*“We have hotline numbers that our clients can use to report any, you know, mis-normal [activities] or challenges or anything that they may not have liked [about VAPN] but also even things that they may have liked about our program.”* (national-Zimbabwe, female, participant 13)
**Yield of VAPN**	**Sensitizing community members & HCWs to VAPN services**	*“This [VAPN] information is also out there as pamphlets that can be obtained and there’s posters that are also available at Ministry of Health facilities.* (national-Zimbabwe, female, participant 13)
	**Using digital tools to support uptake of VAPN services**	*“I know they are piloting it, [an initiative] in Cape Town is using digital [technologies], so using media, to facilitate this [VAPN] process.”* (national-South Africa, male, participant 10)
	**Implementing the Social Network Approach to increase uptake of VAPN services for key populations**	*“So, you could implement [the] social network [approach] based as one wave. So, you say, okay I have got this one client, and I am going to reach out to just this one wave of partners that they give me and that’s the end of it. Or you can have multiple waves where you keep going contact to contact, and the data really does tell us, the more waves you do, the more you are reaching this kind of harder to reach, historically harder to reach groups that really don’t self-identify or have very low contact with the health system”.* (global, female, participant 4)
	**Conducting VAPN training for HCWs** (i.e., peer-based learning)	*“…implementing partners were looking like for the people who can do VPN [voluntary partner notification] really well. And when they spot those people… maybe they take a person from Facility A and then they take that person to another facility like to provide hands-on skills to the people in that facility.”* (community-Tanzania, male, participant 1)
	**Integrating and implementing existing HIV testing strategies to support VAPN services** (i.e., HIV self-testing, mobile testing, and workplace testing)	*“… we did see a number of [studies] using self-testing and showing higher [VAPN] uptake rates.”* (global, female, participant 4)
	**Implementing opt-out VAPN services**	*“We’ve even moved to what we call the 'opt-out approach to increase the [VAPN] uptake.”* (community-Eswatini, female, participant 15)
	**Motivating rural HCWs to provide VAPN services to their own communities**	*“[VAPN works better in more rural areas] because relationships might be a bit more personal, so there's more opportunity to talk… also because generally those nurses have a better… a much stronger feeling with their community that they serve, a much stronger bond. So, [nurses] they because they feel much more connected to that area and sometimes even live in there, I think they're more likely to want to help because it indirectly means helping your community.”* (national-South Africa, male, participant 10)
	**Collaborating between communities and healthcare facilities to support linkage to care**	*“…we had the community linkage approach whereby people who were tested [HIV] positive were also linked to people we call them expert clients.”* (community-Tanzania, male, participant 14)

Uptake of VAPN services was further facilitated through its integration with and scale-up of established HIV testing methods. Some participants described combining VAPN with novel HIV testing models including HIV self-testing (HIVST), mobile testing, and/or workplace testing. Other participants (typically at global level) advised implementation of a so-called “Social Network Approach” to support uptake of HIV testing and VAPN services among key populations. One participant described this process as engaging key populations (e.g., MSM) through repeated testing and outreach *“waves”* (*global, female, participant 4).*

In addition to these methods, a few participants described that their countries increased the uptake (and impact of) VAPN services through: 1) increased training for HCWs (through peer education models), 2) implementation of an opt-out VAPN approach, and 3) keeping HCWs in their own communities. A participant from Eswatini described utilization of an “opt-out” *(community-Eswatini, female, participant 15)* approach which assumed client consent to initiate VAPN services following an HIV-positive result.

Additionally, a national-level participant from South Africa described increasing the uptake of VAPN services in rural areas by keeping rural HCWs in their home communities (as opposed to a previously described solution to rotate rural HCWs) because these HCWs were perceived to display a *“stronger bond*” *(national-South Africa, male, participant 10)* and sense of responsibility towards their own communities.

Thereafter, a participant from Zimbabwe described a strategy to facilitate linkage to HIV services following VAPN implementation through the use of *“expert patients” (national-Zimbabwe, female, participant 11)* who had been previously tested HIV-positive and utilized VAPN services.

### Proposed strategies may enhance future VAPN implementation

In terms of future implementation, proposed courses of action included scale-up of currently practiced facilitators (as outlined in Table 2) as well as additional recommendations to further improve the ethical implementation of VAPN and increase its yield, Table 3. Regarding ethical implementation, some participants suggested to increase multi-sectoral collaboration in VAPN guideline development and implementation. Specifically, a few participants (particularly from Malawi) called for the integration of religious leaders into VAPN service promotion. Other participants at global and national level (Sierra Leone) suggested addressing HIV stigma (and the adverse effects thereof) through the integration of an HIV stigma reduction program into VAPN services. A third suggestion (also from Malawi) was to develop more evidence-based policies designed around findings conducted by independent researchers.

Finally, the participants also suggested four future recommendations to increase VAPN yield. These recommendations included: (1) enhancing digital infrastructure, (2) adapting global VAPN targets, (3) scaling-up family contact tracing services, and (4) displaying VAPN successes to healthcare workers. Specifically, some participants described a need for healthcare facilities to be linked electronically to track index clients and their partners across healthcare facilities, thereby reducing repeat HIV testing and workloads of HCWs. Participants also recommended that future VAPN targets should be adapted at national/community level and the time-frame to reach these targets should be extended. Thereafter, a few participants proposed to increase VAPN yield through the scale-up of family referral systems (FRS). While some participants debated the effectiveness of FRS; other participants described a need to reach family members, particularly untested biological children. Finally, a participant from South Africa described that implementing a *“feedback loop” (national- South Africa, male, participant 10)* could demonstrate to HCWs the impact they have on their communities which could motivate future VAPN service delivery.

**Table 3:** Facilitators proposed by participants to support the implementation of VAPN services in terms of ethical implementation and yield, respectively (*n* = 15)

Facilitator Domains:	Proposed Solutions:	Quotes:
**Ethical Implementation**	**Involving stakeholders to promote VAPN**	*“I think, working with the multi-sectoral partners would also help… So, working with the faith community to enhance partner notification gives you the opportunity to faith leaders to also help address some of the adverse issues that will come about as a result of disclosing or as a result of encouraging one's partner to go for the testing.”* (national-Malawi, male, participant 12)
	**Integrating HIV stigma reduction programs into VAPN services**	*“…by providing programs like VAPN alongside proven anti- or de-stigmatization work would be extremely beneficial… So, I think these sorts of things will be able to provide a more supportive environment for VAPN to happen and to prevent potential negative consequences.”* (global, male, participant 7)
	**Conducting independent research to inform evidence-based policy**	*“I do think that, an independent kind of more scientific approach to operation research and evidence-based policy I think is desperately needed. We have to get out of this monopoly of funding partners to deliver good results.”* (national-Malawi, male, participant 9)
**Yield of VAPN**	**Investing in data infrastructure & simplifying monitoring and evaluation systems**	*“… if there would be a way of identifying the already approached index clients from the HTS [health testing services] to be identified at the ART clinic so that we don’t repeat or we don’t waste time with those already identified at HTS.”* (community-Malawi, male, participant 2)
	**Adapting globally set VAPN targets**	*“I think this issue around targets probably is the most pressing and the targets that are set right now by PEPFAR are driving the agenda, and that probably needs to change.”* (global, female, participant 4)
	**Enhancing & scaling up family HIV contract tracing services**	*“… just to elaborate a little bit on the family [contact tracing] approach. I think it’s quite important because it also takes children into account, and but it looks at an individual more holistically…”* (national-South Africa, male, participant 10)
	**Displaying successes of VAPN to HCWs**	*“… because the feedback loop is generally missing, it’s for me a clear facilitator… But if you can show [ground staff] look, this, this person came because of that [VAPN service] and wouldn't have come [to the health facility] otherwise and look he's on treatment now, she's on treatment now, [it] is a very valuable facilitator.” (national- South Africa, male, participant 10)*

## Discussion

In this multi-level and multi-national qualitative analysis, stakeholders developing and/or implementing HIV VAPN in sub-Saharan Africa identified barriers and facilitators to VAPN implementation. Barriers were identified at global, national, and community levels including: (1) limited independent research; (2) contradictory laws; (3) fragmentation between stakeholder levels; (4) client-level barriers; (5) ethical implications; (6) limited standardization of VAPN reporting; and (7) challenges meeting VAPN targets. Most stakeholders, regardless of implementation level, identified the majority of the barriers at community level, thereby suggesting inefficacy of top-down VAPN guideline recommendation and a greater need for context-specific and tailored VAPN strategies. Stakeholders across implementation levels (particularly among community and national stakeholders) described facilitators they both practiced and proposed to uphold the ethical implementation of VAPN and increase its yield. While some facilitators appeared to contradict one another (i.e., *“opt-out”* VAPN vs. client-centered VAPN); overarchingly, stakeholders seemed to place similar value on enhancing the ethical implementation and yield of VAPN services.

 Our findings suggest that a primary barrier to the implementation of (ethical) VAPN may be attributed to guidelines and targets which are globally and nationally set, as has been found in similar HIV policy research [54, 55]. While global VAPN guidelines are evidence based [1, 3]; the participants in this study reflected that VAPN guidelines and targets were not appropriately tailored to their individual contexts and populations. For example, participants in this study highlighted challenges meeting VAPN targets which they attributed to high target setting, constrained time-frames to meet targets, and financial incentives to reach targets.

To mitigate barriers associated with VAPN policy and target development, we suggest enhancing involvement of local people living with HIV (PLHIV), healthcare workers, and community-level implementers into national and community policy development and implementation strategy sessions [56–58]. We also suggest that key stakeholders are involved in decision making surrounding target setting (e.g., number of persons newly identified as HIV-positive) to ensure that advancement of the UNAIDS 95 targets [12] aligns with SMART (*specific, measurable, achievable, realistic, time-bound*) and human rights frameworks [59–63].

In addition to developing VAPN guidelines which are context-specific, our findings also suggest enhancement of monitoring and evaluation (M&E) systems. As indicated by participants in our study, a major challenge collecting reliable VAPN data (and thereby targets) were use of limited and/or fragmented data systems. Participants in our study described that data system fragmentation and non-standardized data collection methods across facilities led to 1) distorted and duplicated data; 2) challenges collecting and interpreting data; and 3) allowed room for coercion. Our findings reflected similar challenges as to those reported in a recent study from Botswana which proposed that a misclassification of partners as newly identified cases challenged accurate partner notification outcomes [64]. In addition, some participants in our study called attention to severe adverse events (SAEs) they perceived among index clients (including involuntary HIV status disclosure; partnership dissolution; loss of financial security; gender-based violence; or suicide) which were likely under-reported due to non-mandatory SAE reporting systems and/or lack of human resources to carry-out reporting or follow-up of clients. Thus, we recommend VAPN implementing partners to invest in digital infrastructure (e.g., data sharing, storage, and security systems) [65]. We also recommend that M&E systems are co-designed with key stakeholders utilizing the tenants of health equity [66, 67] and human rights [62, 63] to ensure system feasibility, appropriateness, and that VAPN programs are adequately staffed. This may empower communities to manage VAPN implementation, track their progress against targets, and take ownership of VAPN [68]. Monitoring VAPN outcomes (including SAEs) would be beneficial to make timely adaptations to VAPN strategies to ensure ethical VAPN implementation. Similarly, awareness of SAEs may support HCWs to counsel index clients about potential risks associated with VAPN and empower them to make informed decisions about the uptake of VAPN services.

This research also recommends modifications at community-level to engage more index clients into HIV testing and VAPN services. We foremost suggest regular training for healthcare workers and implementing partners on the ethical implementation of VAPN services. This could be conducted through blended learning [40] or peer education models (as implemented by a participant in our study) which have been found to be feasible and acceptable models for training HIV testing skills among HCWs [69]. We additionally suggest reducing client barriers to HIV services through modified healthcare facility opening hours and/or VAPN integration with novel HIV testing methods. For example, VAPN services may benefit from building off of *“moonlight”*^(p.331)^ [70] HIV testing services (i.e., night health services) which has been attributed to increased uptake of HIV testing and decreased stigmatization among vulnerable populations [70, 71]. Similarly, increased HIV testing (and thereby partner notification) may be supported when integrated with HIV self-testing as evidenced by a study in Zambia [72]. Finally, VAPN service delivery may also be supported through integration with telehealth services including online HIV result delivery or virtual partner notification (e.g., SMS, Email) to support individuals unable or unwilling to engage with healthcare facilities [23, 65, 73].

This study is not without limitations. First, this study utilized a purposive sampling method in which authors recruited professional contacts for study participation. The recruitment of participants known to (some of) the authors may have contributed to sampling and/or response biases. However, we aimed to mitigate this challenge by including participants across implementation levels and across various sub-Saharan African countries who likely had varying experiences with VAPN implementation. Second, we did not capture insights from beneficiaries (i.e., index clients) as this was beyond the scope of our study and required additional ethical approval and human resources (e.g., researchers). Including beneficiary perspectives could have triangulated understanding of VAPN implementation barriers and facilitators across all levels and should be considered for future research. Third, due to the study time-frame during the COVID-19 pandemic, interviews and audio recordings were conducted virtually and were at times challenged by internet connectivity and technical difficulties. Finally, as with all qualitative studies, our findings are not generalizable to the views of all VAPN stakeholders in the sub-Saharan region nor the views of all stakeholders within a specific country.

## Conclusion

This multi-level and multi-national qualitative research suggests that the implementation of VAPN for HIV contact tracing in sub-Saharan Africa often follows a top-down cascade and barriers to VAPN implementation are evident at each respective level, the majority of which were identified at community level. Participants in this study mitigated (or expressed desire to mitigate) implementation barriers through various strategies aimed to sustain ethical implementation and increase VAPN yield. Based on our findings, future policies should be built around the barriers and facilitators identified by sub-Saharan African stakeholders. We recommend tailoring VAPN guidelines (and their respective targets) to specific contexts and populations by enhancing involvement of local PLHIV and relevant community stakeholders; addressing structural challenges (e.g., M&E systems); and increasing client engagement through VAPN integration with novel testing strategies (e.g., HIVST). These approaches may maximize the implementation of ethical HIV VAPN services in sub-Saharan African settings and support achievement of the UNAIDS 95–95-95 targets.

### Supplementary Information

**Additional file 1.  **In-depth interview guide.

## Data Availability

The data set (in-depth interviews and descriptive statistics) used and analyzed during this study are available from the corresponding author upon reasonable request.

## Abbreviations

AIDS: Acquired immunodeficiency syndrome
ART: Anti-retroviral therapy
CDC: Centers for Disease Control and Prevention
FRS: Family referral slips
FSW: Female sex worker
HCW: Healthcare worker
HIV: Human Immunodeficiency Virus
HIVST: HIV self-testing
IDI: In-depth interview
IPV: Intimate partner violence
IQR: Inter-quartile range
M&E: Monitoring and evaluation
MSM: Men who have sex with men
NGO: Non-governmental organization
PEPFAR: President’s Emergency Plan for AIDS Relief
PLHIV: People living with HIV
PNS: Partner notification services
PrEP: Pre-exposure prophylaxis
SAE: Severe adverse events
SMART: Specific, measurable, achievable, realistic, time-bound
SSA: Sub-Saharan Africa
TA: Thematic analysis
UNAIDS: Joint United Nations Programme on HIV/AIDS
USAID: United States Agency for International Development
VAPN: Voluntary assisted partner notification
VPN: Voluntary partner notification
WHO: World Health Organization

## Footnotes

1 The UNAIDS 95–95-95 targets build off of the former 90–90-90 targets [19] and aims for 95% of people living with HIV to know their status; 95% of people diagnosed with HIV to receive sustained antiretroviral therapy; and 95% of people receiving antiretroviral therapy to achieve viral suppression by 2030.

2 At the time of this study, laws criminalizing and/or prosecuting HIV transmission were present in all of the countries (at national and community-level) included in this study. These countries included Kenya, Zimbabwe, Sierra Leone, Tanzania, Malawi, South Africa, and Eswatini [52].

3 At the time of this study, Kenya, Zimbabwe, Sierra Leone, Tanzania, Malawi, and Eswatini had laws criminalizing same-sex acts (through imprisonment or through undefined penalties) [52].

4 At the time of this study, all countries (at national and community-level) had laws criminalizing sex work [52]. Some of these countries faced challenges with police violence against female sex workers during the time of this study [53].
