# Supplementary material for: “The targets…are driving the agenda and that probably needs to change”: stakeholder perspectives on HIV partner notification in sub-Saharan Africa
Source: BMC Public Health. 2024 Feb 19;24:521. doi: 10.1186/s12889-023-17422-9 (PMC10877856; doi:10.1186/s12889-023-17422-9)
Supplement: Supplementary file 1 — Additional file 1. In-depth interview guide. [file 12889_2023_17422_MOESM1_ESM.docx]

**Additional file 1: In-Depth Interview Guide**

**Good [afternoon/morning] thank you for participating today**!

The purpose of this project is to develop a better understanding of voluntary assisted partner notification from the perspective of stakeholders, policy makers and implementers working in countries where VAPN is being implemented.

I have asked you to meet with me in the hopes of learning more about your personal opinions and experiences with VAPN in terms of the barriers and facilitators to implementation, perceptions around rights and disclosure and opportunities for improvement.

We will be taking notes and also recording our conversation so that we can accurately capture and report your views. Your comments will be combined with those from other interviews.

| **Global Level** | **National Level** | **Community Level** |
| --- | --- | --- |
| 1. **Please tell me a little bit about yourself. What is your current role?** 2. *PROBE:* For how long have you worked in the field of HIV? 3. **Can you tell me something about the global implementation of VAPN?** 4. **Can you tell me something about the implementation in sub-Saharan African countries?** 5. *PROBE:* What incentives are provided to countries for implementing the VAPN recommendations (Incentives to train additional staff, cost/time needed to locate partners etc.) 6. **Please tell me about the information available for VAPN.** 7. *PROBE:* What information is available for implementers? 8. *PROBE:* What information is available for clients? 9. *PROBE*: What information is available to the general public? 10. *PROBE:* Where do you see information gaps? 11. **How do you monitor implementation of the 2016 WHO VAPN recommendation?** 12. *PROBE:* Please tell me about the reporting guidelines for VAPN implementation. 13. **What would make VAPN cost-effective in your opinion?** 14. *PROBE:* Which factors need to be considered for cost-effectiveness estimates) 15. **Which factors have facilitated VAPN implementation in sub-Saharan Africa in your opinion?** 16. **Which factors do you think have proved challenging for VAPN implementation in sub-Saharan Africa?** 17. **What is currently known about the outcomes of VAPN?** 18. *PROBE:* What outcomes have you seen in terms of the HIV epidemic? 19. *PROBE:* Please tell me about any positive outcomes you can think/know of. 20. *PROBE*: Please tell me about any negative outcomes you can think/know of. 21. *PROBE:* How are these outcomes measured? 22. *PROBE:* Has VAPN had any effect on yield (identifying more HIV-positive people)? 23. **What do you think are the human-rights considerations of VAPN?**   a) *PROBE:* What is the programmatic guidance for maintaining the voluntary nature of VAPN?  b) *PROBE:* What is the programmatic guidance for maintaining confidentiality and unintended disclosure?  c) *PROBE:* What are the guidelines for preventing and addressing adverse effects (i.e. Intimate Partner Violence)?  d) *PROBE:* How do you think implementation compares to the guidelines?  e) *PROBE:* What adverse human-rights effects have you been made aware of in the context of VAPN?   1. **Do you think any changes are needed in implementing VAPN?** 2. *PROBE:* What do you think can be done to enhance the experience of clients guided to VAPN? 3. *PROBE*: What could enhance the experience of partners of index-clients? 4. **Can you tell me about any other methods of partner notification which you believe to be preferable to VAPN and why?** 5. **Please tell me about any other thoughts you have regarding VAPN.** 6. **Is there anything else you would like to add? Any questions that I should have asked you?** | 1. **Please tell me a little bit about yourself. What is your current role?** 2. *PROBE:* For how long have you worked in the field of HIV? 3. **When did your country start to get involved with VAPN?** 4. *PROBE:* Can you take me through the process of introducing VAPN as a national strategy? 5. *PROBE:* How have you translated global VAPN goals to a national policy? 6. *PROBE:* How have you translated the national VAPN policy to the community level? 7. *PROBE:* How was VAPN introduced at the community level? (national simultaneous introduction, stepwise/phased introduction) 8. *PROBE:* How important is VAPN in the overall HIV testing strategy? 9. *PROBE:* What is the national coverage of VAPN? 10. *PROBE:* How many provinces/counties are covered? 11. *PROBE:* Who are the main implementers (NGOs, Health facilities…) 12. **Please tell me about any incentives there are for those centers implementing the VAPN recommendation?** (probe in terms of manpower, time and cost) 13. **What is the estimated cost of implementing VAPN?** 14. **Do you have reporting guidelines for VAPN?** 15. *PROBE*: How do those implementing VAPN at the community level report VAPN to you? 16. *PROBE:* How do you report country-level findings to global actors (i.e. WHO, CDC)? 17. **How is VAPN conducted in communities with small populations?** 18. **What do you see as facilitators to implementation of the VAPN recommendation?** 19. **What do you see as barriers to the implementation of the VAPN recommendation?** 20. **What do you believe that outcomes of VAPN are?** 21. *PROBE:* Have you recognized any positive effects? 22. *PROBE:* How do you measure these effects? 23. *PROBE:* Have you recognized any adverse effects? 24. *PROBE:* How do you measure these effects? 25. *PROBE:* Can you say anything about the yield of VAPN so far? 26. **How do you view VAPN through a human-rights point of view?** 27. *PROBE:* What is the programmatic guidance for maintaining the voluntary nature of VAPN? 28. *PROBE:* What is the programmatic guidance for maintaining confidentiality and unintended disclosure? 29. *PROBE:* What are the guidelines for preventing and addressing adverse effects (i.e. Intimate Partner Violence)? 30. *PROBE:* How does implementation compare with the guidelines? 31. *PROBE:* Please describe any incidences where you have noticed or heard of adverse human-rights effects in the context of VAPN. 32. **Where do you see opportunities for improvement of VAPN?** 33. *PROBE:* Policy level 34. *PROBE:* Implementation / Clinic level 35. *PROBE*: Experiences of clients 36. *PROBE:* Experience of partners of index-client 37. **Can you tell me about any other methods of partner notification which you believe to be preferable to VAPN and why?** 38. **Please tell me about any other thoughts you have regarding VAPN.** 39. **Is there anything else you would like to add? Any questions that I should have asked you?** | 1. **Please tell me a little bit about yourself. What is your current role?** 2. *PROBE:* For how long have you worked in the field of HIV?   **2. How was VAPN introduced at community level?**   1. *PROBE:* How would you describe the process of introducing VAPN as part of the HIV testing strategy? 2. *PROBE:* Did you receive training on how to communicate VAPN? 3. *PROBE:* How were the communities informed about the implementation of VAPN? 4. *PROBE:* How were national organizations (i.e. National Association for People Living with HIV and AIDS) informed about the implementation of VAPN? 5. *PROBE:* What are your thoughts about this process? 6. *PROBE:* How do you report VAPN implementation findings to the country level? 7. *PROBE:* What costs occur at implementation level for VAPN? (optional, depending on interviewee)   **3.What do you believe facilitates the implementation of VAPN?**  **4.What do you believe hinders the implementation of VAPN?**  **5.What do you believe the outcomes of VAPN have been?**   1. *PROBE:* Have you recognized any positive effects? 2. *PROBE:* How do you measure these effects? 3. *PROBE:* Have you recognized any adverse effects? 4. *PROBE:* How do you measure these effects? 5. *PROBE:* What yield have you noticed, if any? 6. *PROBE:* What do you think the uptake of VAPN has been in those you have recommended it to?   **6.Could you take me through the process of VAPN?**   1. *PROBE:* How do you ask clients to provide contact information for sexual partners? 2. **Please walk me through an example where a client has had a positive experience with VAPN.** 3. *PROBE:* What made it positive? 4. *PROBE:* Why do you remember this particular example? 5. **Please walk me through an example of where a client had a negative experience with VAPN**. 6. *PROBE:* What made it negative? 7. *PROBE:* Why do you remember this particular example? 8. **How do you view VAPN from a human-rights perspective?** 9. *PROBE:* What guidelines do you follow to maintain the voluntary nature of VAPN? 10. *PROBE:* What guidelines do you follow to maintain confidentiality and disclosure? 11. *PROBE:* What guidelines do you follow to prevent or address adverse effects (i.e. IPV)? 12. *PROBE:* How does actual implementation compare with the guidelines? 13. **Where do you see opportunities for improvement of VAPN?** 14. *PROBE:* Policy level 15. *PROBE:* Implementation / Clinic level 16. *PROBE:* Experiences of clients 17. *PROBE:* Experience of partners of index-client 18. **Can you tell me about any other methods of partner** **notification which you believe to be preferable to VAPN and why?** 19. **Please tell me about any other thoughts you have regarding VAPN.** 20. **Is there anything else you would like to add? Any questions that I should have asked you?** |
